# Mycobacterium tuberculosis Toxin CpnT Is an ESX-5 Substrate and Requires Three Type VII Secretion Systems for Intracellular Secretion

**DOI:** 10.1128/mBio.02983-20

**Published:** 2021-03-02

**Authors:** B. Izquierdo Lafuente, R. Ummels, C. Kuijl, W. Bitter, A. Speer

**Affiliations:** a Section of Molecular Microbiology, Amsterdam Institute of Molecular and Life Sciences (AIMMS), Vrije Universiteit Amsterdam, Amsterdam, The Netherlands; b Department of Medical Microbiology and Infection Control, Amsterdam UMC, Location VU Medical Center, Amsterdam, The Netherlands

**Keywords:** *Mycobacterium*, *Mycobacterium tuberculosis*, exotoxins, protein secretion, secretion systems

## Abstract

CpnT, a NAD^+^ glycohydrolase, is the only known toxin that is secreted by Mycobacterium tuberculosis. CpnT is composed of two domains; the C-terminal domain is the toxin, whereas the N-terminal domain is required for secretion. CpnT shows characteristics of type VII secretion (T7S) substrates, including a predicted helix-turn-helix domain followed by a secretion motif (YxxxE). Disruption of this motif indeed abolished CpnT secretion. By analyzing different mutants, we established that CpnT is specifically secreted by the ESX-5 system in Mycobacterium marinum under axenic conditions and during macrophage infection. Surprisingly, intracellular secretion of CpnT was also dependent on both ESX-1 and ESX-4. These secretion defects could be partially rescued by coinfection with wild-type bacteria, indicating that secreted effectors are involved in this process. In summary, our data reveal that three different type VII secretion systems have to be functional in order to observe intracellular secretion of the toxin CpnT.

## Abstract (precis)

CpnT, a NAD^+^ glycohydrolase, is the only known toxin that is secreted by Mycobacterium tuberculosis. CpnT is composed of two domains; the C-terminal domain is the toxin, whereas the N-terminal domain is required for secretion.

## INTRODUCTION

Mycobacterium tuberculosis is a facultative intracellular pathogen and the causative agent of tuberculosis (TB). When infecting the host, M. tuberculosis is internalized by immune cells, primarily macrophages (1–3). One of the mechanisms by which M. tuberculosis is able to survive and proliferate within these infected cells is by permeating the phagosomal membrane using secreted effector proteins (4). Also, other secreted mycobacterial virulence factors manipulate the host cell defenses and allow the bacterium to adapt to the hostile macrophage environment (2, 5).

In order to secrete virulence proteins across the cell envelope, didermic bacterial pathogens have developed specific secretion systems that differ from the general secretion pathways (2, 5–7). In the genus of mycobacteria, secretion across the inner and outer membrane is mediated by a specialized secretion system, the type VII secretion system (T7S), also named the ESX secretion system (8–10). The genome of M. tuberculosis encodes five of these ESX secretion systems, ESX-1 to ESX-5 (9, 11, 12). Several types of substrates are secreted by the T7S, the Esx, ESP, PE, and PPE proteins (9–11, 13, 14), all belonging to the EsxAB family. Usually, these substrates are secreted as a dimer, in which one of the partners has an N-terminal helix-turn-helix motif followed by the sequence motif YxxxD/E, required for export (9, 15).

The ESX-1 secretion system is required for full pathogenicity and is responsible for the secretion of a set of crucial virulence factors that play a key role in the escape of mycobacteria from the phagosome to the cytosol during infection (16–18). Evolutionary analysis indicates that the ESX-5 system is the most recently evolved system, and surprisingly, it is only present in slow-growing pathogenic mycobacteria (6, 19). ESX-5 is an essential secretion system required for nutrient uptake and membrane permeability (20). Moreover, it secretes a vast number of substrates, including most PE and PPE proteins, which play a role in the host immunomodulation and immune evasion (21, 22). ESX-3 is necessary for iron and zinc acquisition and therefore is essential for bacterial growth (23–25). Little has been reported about the function of ESX-4 in M. tuberculosis. In the nonpathogenic Mycobacterium smegmatis, ESX-4, in combination with ESX-1, modulates DNA transfer by a special form of chromosomal conjugation (26, 27). Recently, a study showed that, in the fast-growing pathogen Mycobacterium abscessus, ESX-4 participates in phagosomal rupture and contributes to intracellular survival (28).

Toxins are a major class of virulence factors, and they induce direct damage to the infected host cells (29). Mycobacteria were believed to lack classical exotoxins, since no known bacterial toxin could be identified (30, 31). This hypothesis was refuted by the identification of CpnT (Rv3903c), the first characterized exotoxin of M. tuberculosis (32). CpnT is a two-domain protein in which the toxic activity resides in the C-terminus. Inside macrophages, M. tuberculosis releases the C-terminal domain, which efficiently hydrolases the essential NAD^+^ coenzyme, resulting in a necrotic type of cell death (33, 34). This domain is thus called tuberculosis necrotizing toxin (TNT). Since the NAD^+^ coenzyme is also present and essential in prokaryotic cells, M. tuberculosis protects itself by producing an antitoxin known as the immunity factor for TNT (IFT). This antitoxin is encoded by the adjacent gene and inhibits the toxic domain of CpnT by direct interaction (34). The N-terminus of CpnT was suggested to function as a pore-forming domain required for secretion, nutrient uptake, and antibiotic susceptibility (32, 35). Secretion of this toxin is still poorly understood. In this study, we identify CpnT as a new type VII substrate of pathogenic mycobacteria.

## RESULTS

### Identification of CpnT toxin as an ESX substrate.

**FIG 1:**
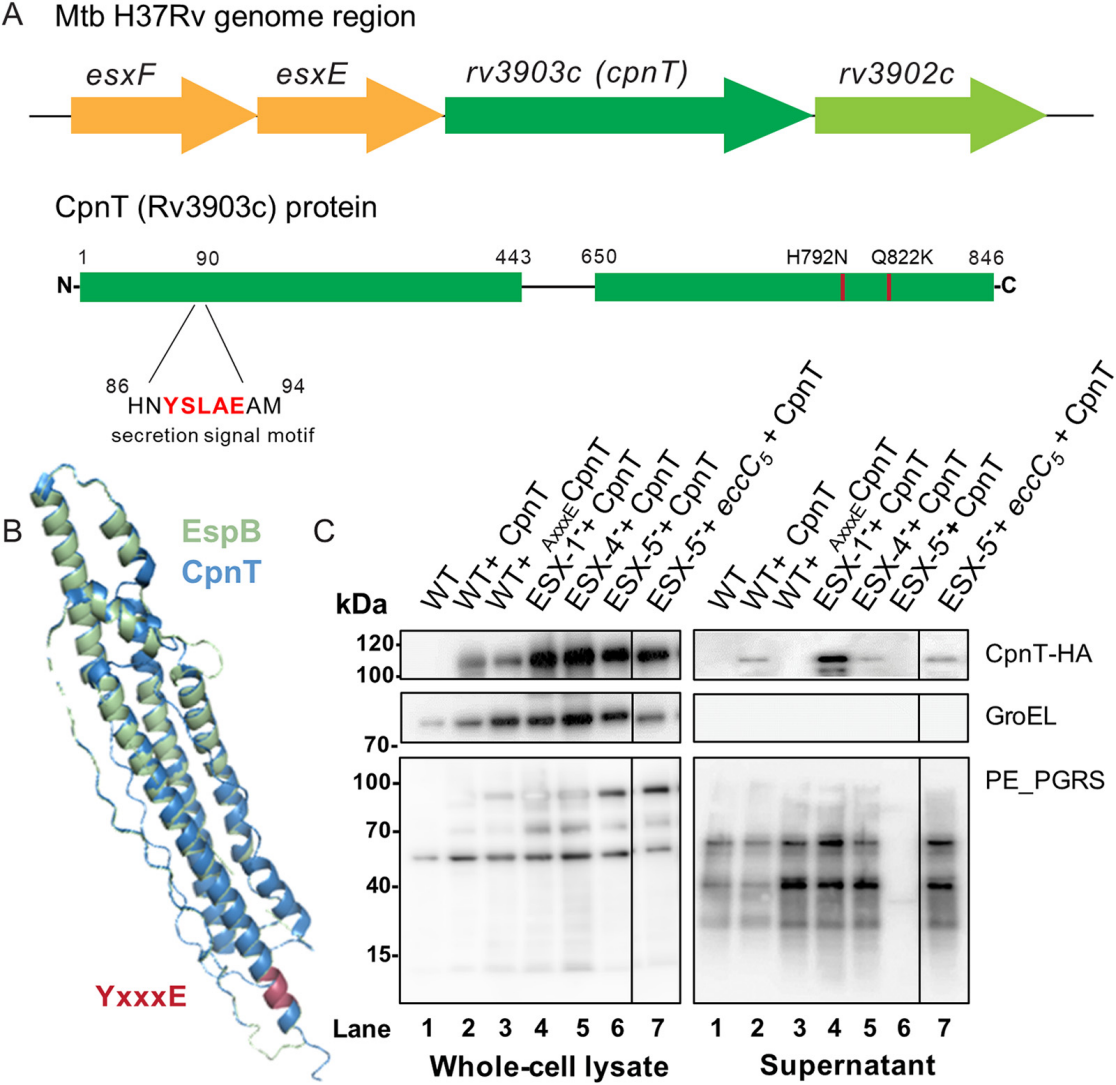
CpnT is a type VII substrate secreted in an ESX-5-dependent manner. (A) Genetic organization of the *esxF-esxE-cpnT-rv3902c* (*IFT*) operon and CpnT domain organization. (B) Structural modeling of CpnT with the N-terminus of EspB as a template (PDB accession number 4WJ2; M. smegmatis); confidence of 83% of the N-terminus domain (amino acids 48 to 276). The N-terminus of EspB is displayed in blue, and CpnT in light green; the secretion signal motif is marked in red (YxxxE). Root-mean-square deviation (RMSD) for the structural alignment is 1.57 Å (402 atoms out of 972 aligned atoms). (C) Secretion analysis of M. marinum strains, WT, ESX-1-deficient (ESX-1^−^, M^VU^), ESX-4-deficient (ESX-4^−^, Δ*eccC_4_*), and ESX-5-deficient (ESX-5^−^, Δ*eccC_5_ + mspA*) expressing CpnT-HA or WT expressing _Axxxe_CpnT-HA. Whole-cell lysate and supernatant preparations were immunoblotted for CpnT detection (anti-HA antibody); immunodetection of GroEL was used as the lysis control, and PE_PGRS, as a control for efficient secretion. Complementation of the ESX-5-deficient strain (ESX-5^–^ + *eccC_5_*, Δ*eccC_5_ + eccC_5_*) restored PE_PGRS secretion.

While it has been well established that many human pathogens utilize toxins to defend themselves against host cells, it is only a recent discovery that the genome of M. tuberculosis, one of the oldest known and most devastating human pathogens, encodes a secreted toxin (32). CpnT is secreted across the inner and outer membranes of the bacterium and released into the cytosol of the infected phagocytic cells to cause necrotic cell death (34). However, the mechanism of secretion and its involved secretion system remained unknown. We observed that CpnT does not have a classical signal sequence but contains a putative type VII secretion motif at the N-terminus, 88YxxxE (Fig. 1A). Using Phyre2 homology modeling software, we identified that the N-terminal domain of CpnT (amino acids 0 to 443) is predicted with more than 95% confidence to fold similarly to several ESX substrates (36). Furthermore, the highest coverage (amino acids 48 to 276) was observed with the N-terminal helical domain of EspB of Mycobacterium smegmatis (Fig. 1B). ESX substrate genes are often clustered in the genome and/or located within the ESX loci (9). Interestingly, we observed two unique *esxA*-type genes, *esxF* and *esxE*, upstream of the *cpnT* gene and its respective antitoxin (*rv3902c*) (Fig. 1A, Fig. S1). Taking those findings into account, we hypothesized that CpnT is a T7S substrate.

10.1128/mBio.02983-20.1 FIG S1 The EsxF and EsxE structures are similar to the EsxA structure in M. tuberculosis. Structural modelling of EsxF and EsxE using the template EsxA from M. tuberculosis. EsxA is displayed in light blue, esxF in light yellow, and EsxE in orange. The root-mean-square deviation (RMSD) for both structural alignments is 1.47 Å (393 atoms out of 393 aligned atoms). Download FIG S1, JPG file, 0.5 MB. Copyright © 2021 Izquierdo Lafuente et al. 2021 Izquierdo Lafuente et al. https://creativecommons.org/licenses/by/4.0/ This content is distributed under the terms of the Creative Commons Attribution 4.0 International license.

In order to test our hypothesis, we first tried to express the M. marinum orthologue of *cpnT* (*mmar_5464*) modified with an HA-tag in the cognate parent strain M. marinum M^USA^. However, we could not observe any expression (data not shown). Next, we tried CpnT of M. tuberculosis. We amplified the operon *esxF-esxE-cpnT-rv3902c* and expressed it under the control of the constitutive promoter p_imyc_. The C-terminus of CpnT was translationally fused to an HA-tag for subsequent immunological detection. The toxin domain of CpnT encodes a NAD^+^ glycohydrolase, which makes the toxin universally lethal to both prokaryotic and eukaryotic cells (32). Since we exclusively wanted to investigate CpnT transport without risking creating artifacts caused by the nonsecreted toxin, we introduced two point mutations in *cpnT*, resulting in amino acid substitutions H792N and Q822K, which have been previously described to abolish CpnT toxicity (34). In all our experiments we are using this detoxified version of CpnT. The M. marinum wild-type (WT) strain was transformed with the *cpnT* construct, and secretion analysis was performed by precipitation of the supernatant proteins. To examine whether CpnT was a T7S substrate, we also modified the tyrosine residue of the potential secretion signal motif (YxxxE) of CpnT, resulting in _AxxxE_CpnT (Y88A). When analyzing culture supernatants of WT producing CpnT or _AxxxE_CpnT, we observed that CpnT was secreted in modest amounts by WT bacteria (Fig. 1C, lanes 1 to 3, supernatant). The intracellular protein control (GroEL) was only observed in the pellet fraction (whole-cell lysate), confirming that the presence of CpnT in the supernatant was not due to cell lysis. Interestingly, we only observed the full-length version of the CpnT protein (∼100 kDa) and not a processed band (∼26 kDa) as reported by Danilchanka et al. (31), suggesting that this processing is specific for M. tuberculosis. Importantly, the secretion of CpnT was abolished in WT bacteria expressing the secretion mutant _AxxxE_CpnT (Fig. 1C, lane 3, supernatant). This reduction in secretion was not accompanied by an increase of intracellular amounts of CpnT, a characteristic that is also observed for other T7S substrates in M. marinum (15). As an additional control, we performed a double-filter colony blot, as described in Abdallah et al. (8). WT+ CpnT and WT+ _AxxxE_CpnT were grown on nitrocellulose filters as a control for proper protein diffusion, and immune detection served a WT strain containing the HA-tagged EsxA substrate (37), which is secreted via ESX-1. The double colony blot assays confirmed our secretion analysis by showing that CpnT was secreted in the WT but not when the T7S motif was mutated (Fig. S2). These experiments show that CpnT of M. tuberculosis is secreted by M. marinum and that residue Y88 plays an important role in this process, indicating that CpnT is probably a T7S substrate.

10.1128/mBio.02983-20.2 FIG S2 CpnT secretion depends on the ESX-5 secretion system. Double filter colony blot analysis of M. marinum WT, ESX-1- (M^VU^), ESX-4- (Δ*eccC_4_*), and ESX-5-deficient (Δ*eccC_5_ + mspA*) strains expressing CpnT-HA or WT expressing _AxxxE_CpnT-HA. WT containing EsxA-HA was used as a positive control for secretion. Colonies were grown on top of a nitrocellulose filter. When colonies were visible, the filter was transferred on top of a new filter and grown for 2 days before the secreted proteins were detected by Western blot analysis. CpnT-HA and EsxA-HA were detected using an anti-HA antibody. EsxA antibody was used as secretion control. Download FIG S2, JPG file, 0.6 MB. Copyright © 2021 Izquierdo Lafuente et al. 2021 Izquierdo Lafuente et al. https://creativecommons.org/licenses/by/4.0/ This content is distributed under the terms of the Creative Commons Attribution 4.0 International license.

### CpnT localizes to the bacterial surface during macrophage infection.

**FIG 2:**
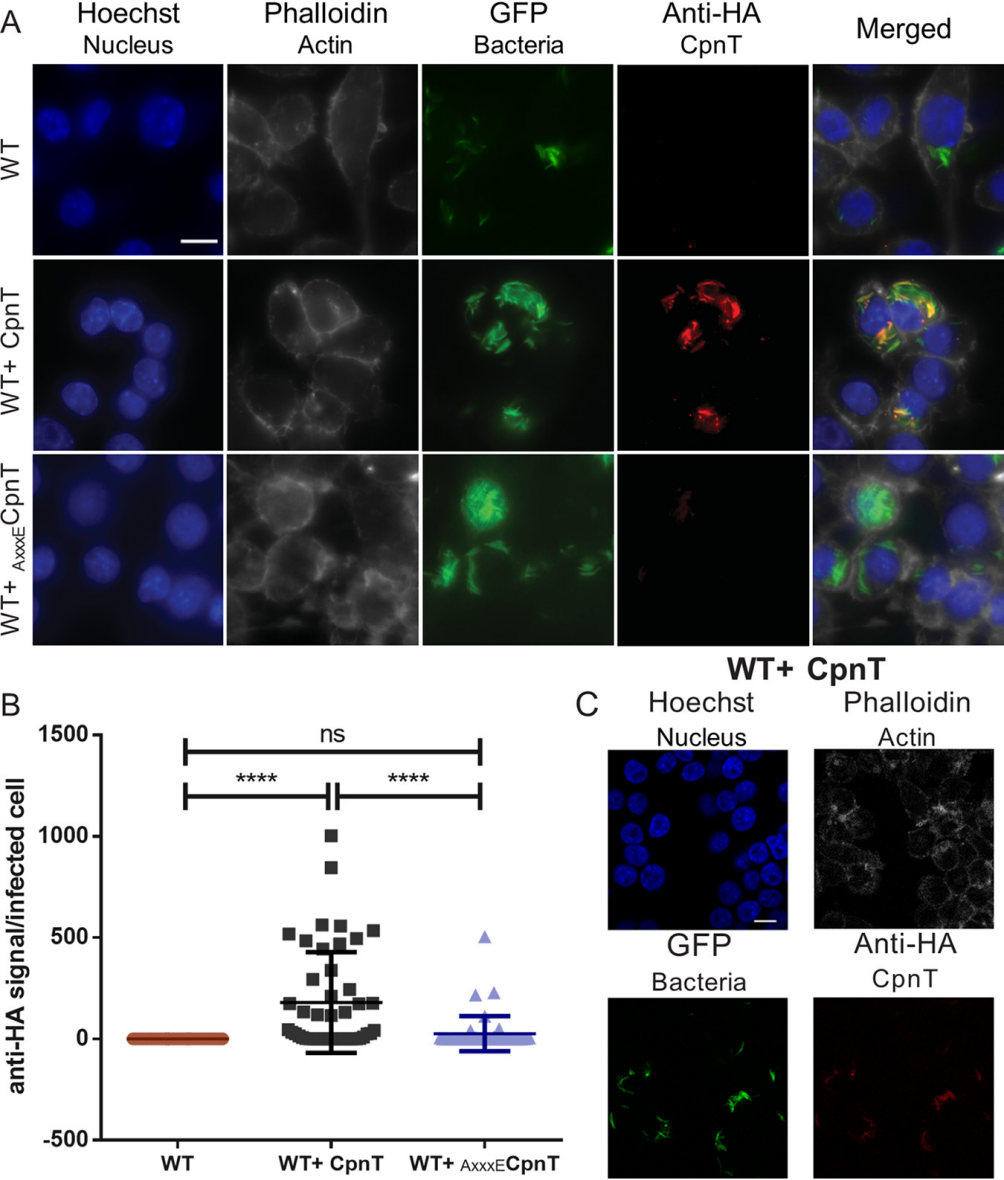
Surface localization of CpnT within macrophages. (A) Fluorescence microscopy of infected RAW 264.7 macrophages with WT, WT+ CpnT, and WT+ _AxxxE_CpnT. All bacterial strains carried L5::*gfp* for fluorescence detection. Macrophages were infected at an MOI of 2 and fixed at 24 h postinfection. Cells were permeabilized with 0.2% Triton X-100 and immuno-labeled with anti-HA antibody (CpnT-HA). Additional dyes were used to label the nuclei (Hoechst) and the actin filaments (Phalloidin). The scale bar represents 10 μm. Images are a representation of five biological replicates. (B) The fluorescence quantification of the anti-HA signal per infected RAW macrophage was calculated using automated image analysis software (CellProfiler). Analysis was performed on one representative experiment out of three independent experiments, with a total number of 40 to 46 infected macrophages. One-way ANOVA and multiple comparisons using Dunnet’s statistical test were performed for statistical significance. ns, *P* > 0.05; ****, *P* < 0.0001. (C) Confocal microscopy of macrophages infected with WT+ CpnT strain. Infection conditions and labeling procedure were identical to those for panel A. The scale bar represents 10 μm.

Next, we wanted to determine whether CpnT was also secreted by M. marinum within macrophages, since CpnT is a virulence factor secreted during infection (34). We infected RAW 264.7 macrophages with the WT bacteria expressing *cpnT* or *_AxxxE_cpnT*. These bacteria also expressed *gfp* from an L5-based integrative vector. Then, 24 h postinfection, cells were permeabilized and immunolabelled with anti-HA antibody to detect the HA-tag on CpnT. Additional dyes were used to label the nuclei and the actin filaments in order to define the eukaryotic cells. Cells were imaged by fluorescence microscopy, and the anti-HA signal (CpnT) of the infected macrophages was quantified (Fig. 2A). Interestingly, when infecting the macrophages with WT bacteria expressing *cpnT*, CpnT did mostly colocalize with the bacteria, suggesting a bacterial surface localization (Fig. 2A).

Quantification of these results showed that only 61% of the infected macrophages showed detectable levels of secreted CpnT (Fig. 2B). These differences between individual infected macrophages could be resulting from differences in the infection stage of each macrophage and attributed to the heterogenic nature of mycobacterial infections (38).

The point mutation in the secretion signal (Y88A) abolished CpnT secretion in culture, but during infection, we could observe some macrophages that were positive for CpnT-HA. However, the amount of detected _AxxxE_CpnT was minimal compared to the control.

To corroborate CpnT localization inside the macrophage, confocal microscopy was used to image the cells with a high accuracy in focal depth (Fig. 2C). CpnT was exclusively detected on the surface of the bacteria, thus confirming the previous observations. Again, the CpnT signal was highly variable, with some cells showing high fluorescence, whereas others were negative (data not shown).

### EsxE and EsxF are required for CpnT secretion.

**FIG 3:**
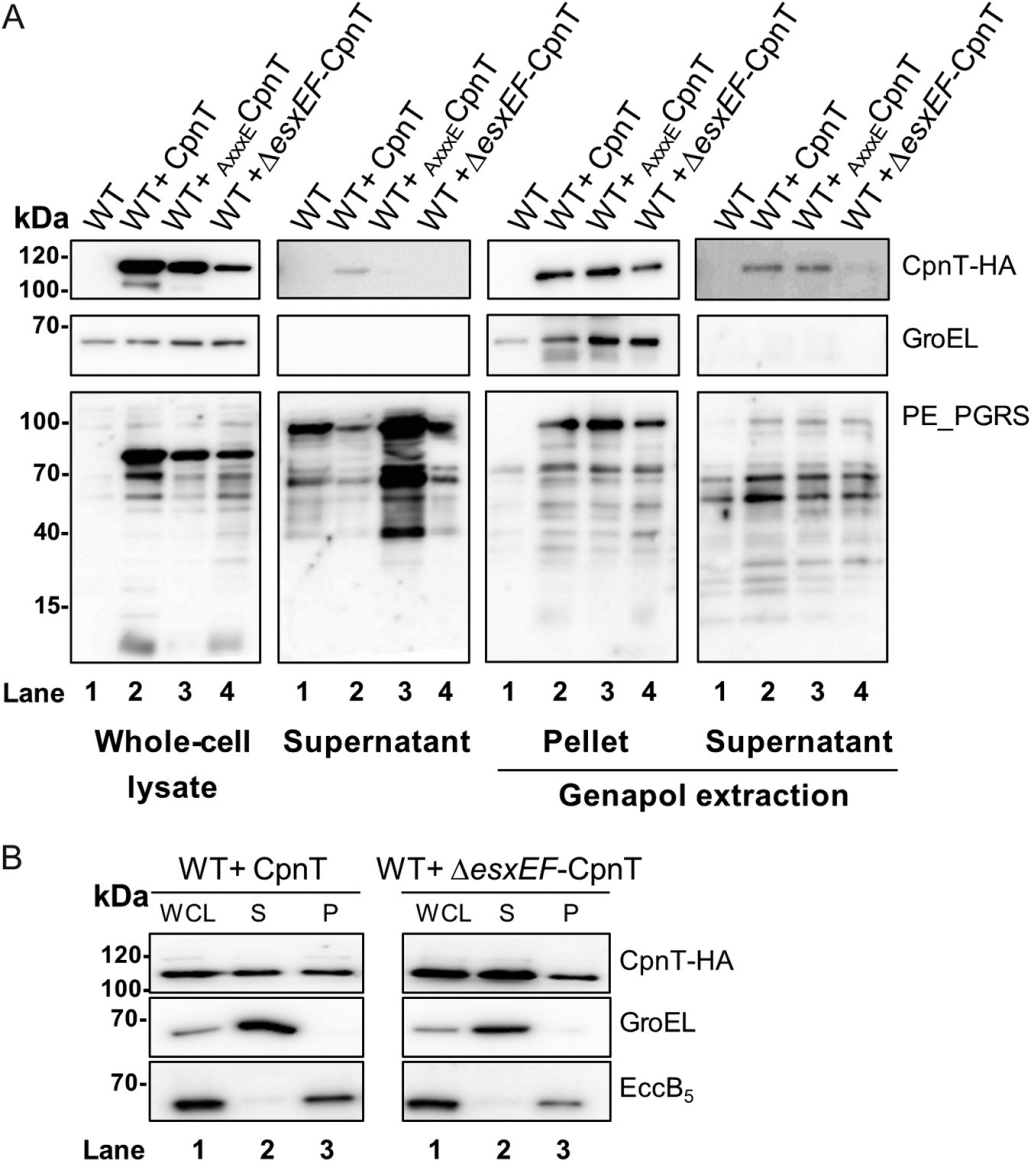
EsxE and EsxF are required for CpnT secretion. (A) Secretion analysis of WT+ CpnT, _AxxxE_CpnT, and Δ*esxEF*-CpnT strains. Whole-cell lysate (WCL) and supernatant preparations were immunoblotted for CpnT detection (anti-HA antibody); immunodetection of GroEL was used as a lysis control, whereas PE_PGRS was used as a control for secretion. Surface proteins were extracted from intact cells with the detergent Genapol X-080 (Genapol supernatant); nonextracted proteins remained in the Genapol pellet fraction. GroEL was used as a cytosolic control, whereas PE_PGRS was used as a control for protein extraction. (B) Subcellular fractions of WT+ CpnT and WT+ Δ*esxEF*-CpnT strains were obtained using differential centrifugation steps. Whole-cell lysate (WCL), soluble proteins (S), and pellet fraction (P, insoluble proteins) were collected. Detection of GroEL (cytosolic protein) and EccB_5_ (membrane-associated protein) with specific antibodies was used as a control for the supernatant and pellet fraction, respectively.

Secretion of different ESX-1 substrates is dependent on the presence of the EsxAB pair (9, 39, 40). Because CpnT is located in the same operon with two *esx-*like genes, *esxE* and *esxF*, we studied whether secretion of CpnT was dependent on the presence of these Esx proteins. We performed a secretion analysis of WT+ CpnT and WT+ Δ*esxEF*-CpnT. As a control, we used WT strains containing _AxxxE_CpnT (Fig. 3A). As observed before, CpnT could only be secreted when the motif secretion signal was intact (YxxxE). As expected, CpnT secretion was also completely abolished when *esxE* and *esxF* were absent, suggesting that CpnT secretion is dependent on the EsxE-EsxF protein pair. Again, this secretion defect was not linked to an accumulation of CpnT in the cell pellet fraction.

We observed that both mutant constructs, the Δ*esxEF*-CpnT and the _AxxxE_CpnT, could not be secreted into the culture supernatant of WT M. marinum. We aimed to investigate further the subcellular localization of these constructs by extraction of bacterial surface proteins using the mild detergent Genapol X-080 (41) (Fig. 3A, Genapol extraction). Our experiment determined that part of CpnT in WT bacteria was surface-localized and could be extracted by detergent. Surprisingly, WT+ _AxxxE_CpnT also showed the corresponding 100-kDa band of CpnT in the Genapol supernatant in similar amounts, suggesting that this mutation did not completely block the surface localization of CpnT. However, CpnT was not detected in the WT+ Δ*esxEF*-CpnT strain, indicating that the EsxEF protein pair is essential for secretion and surface localization.

In addition, we performed a subcellular fractionation on these strains by separating the bacteria into whole-cell lysate (WCL), soluble protein (S), and insoluble membrane protein (pellet [P]) fractions (Fig. 3B). GroEL protein was used as a control for the soluble protein fraction, and EccB_5_, a membrane protein of the ESX-5 machinery complex, was used as the membrane control. Notably, although CpnT was present in both the soluble fraction and the pellet fraction, the absence of *esxEF* did not change subcellular localization.

### CpnT protein is secreted in an ESX-5-dependent manner.

After showing that CpnT is probably a T7S substrate, we continued to investigate which ESX system is involved in its secretion. We therefore transformed the CpnT expression construct in a set of M. marinum strains lacking one specific functional ESX system. The M^VU^ strain, designated ESX-1 deficient (ESX-1^–^), is lacking ESX-1 secretion due to a spontaneous frameshift mutation in the *eccCb_1_* gene (5). We constructed a mutant of ESX-4 by deleting *eccC_4_* using homologous recombination (ESX-4 deficient or ESX-4^–^). The Δ*eccC_5_* knockout strain, ESX-5-deficient strain (ESX-5^–^), was obtained in the presence of the porin MspA to negate ESX-5 essentiality, as previously described (20). The ESX-2 system is not found in the genome of M. marinum (40) and therefore was not further studied. ESX-3 is required for iron uptake and was shown to be essential for *in vitro* growth (42, 43). A study of M. tuberculosis showed that deletions in ESX-3 could be obtained by supplementing the medium with the alternative iron source hemin and Tween 80 (41). However, similar deletion attempts in our lab using M. marinum were unsuccessful (data not shown), and therefore an ESX-3 secretion mutant was not included. We produced whole-cell lysates and collected culture supernatants of the three different ESX-deficient strains expressing *cpnT* and performed Western blot analysis (Fig. 1C).

In all tested strains (ESX-1^–^, ESX-4^–^, and ESX-5^–^) CpnT was successfully produced and present in the whole-cell lysates (Fig. 1C, lanes 4 to 6, whole-cell lysate). In the ESX-1^–^ and ESX-4^–^ strains, CpnT was also present in the culture supernatant (Fig. 1C, lanes 4 and 5 supernatant). The exception was the Δ*eccC_5_* (ESX-5^–^) deletion strain, which did not show any CpnT in the culture supernatant (Fig. 1C, lane 6, supernatant). Double colony blot assays confirmed this observation (Fig. S2). Moreover, when the ESX-5 secretion mutant was complemented with an intact *eccC_5_* gene copy (ESX-5^–^ + *eccC_5_*), CpnT secretion was restored, confirming that secretion of CpnT in M. marinum is dependent on a functional ESX-5 system (Fig. 1C, lane 7, supernatant).

### CpnT secretion depends on three secretion systems during macrophage infection.

**FIG 4:**
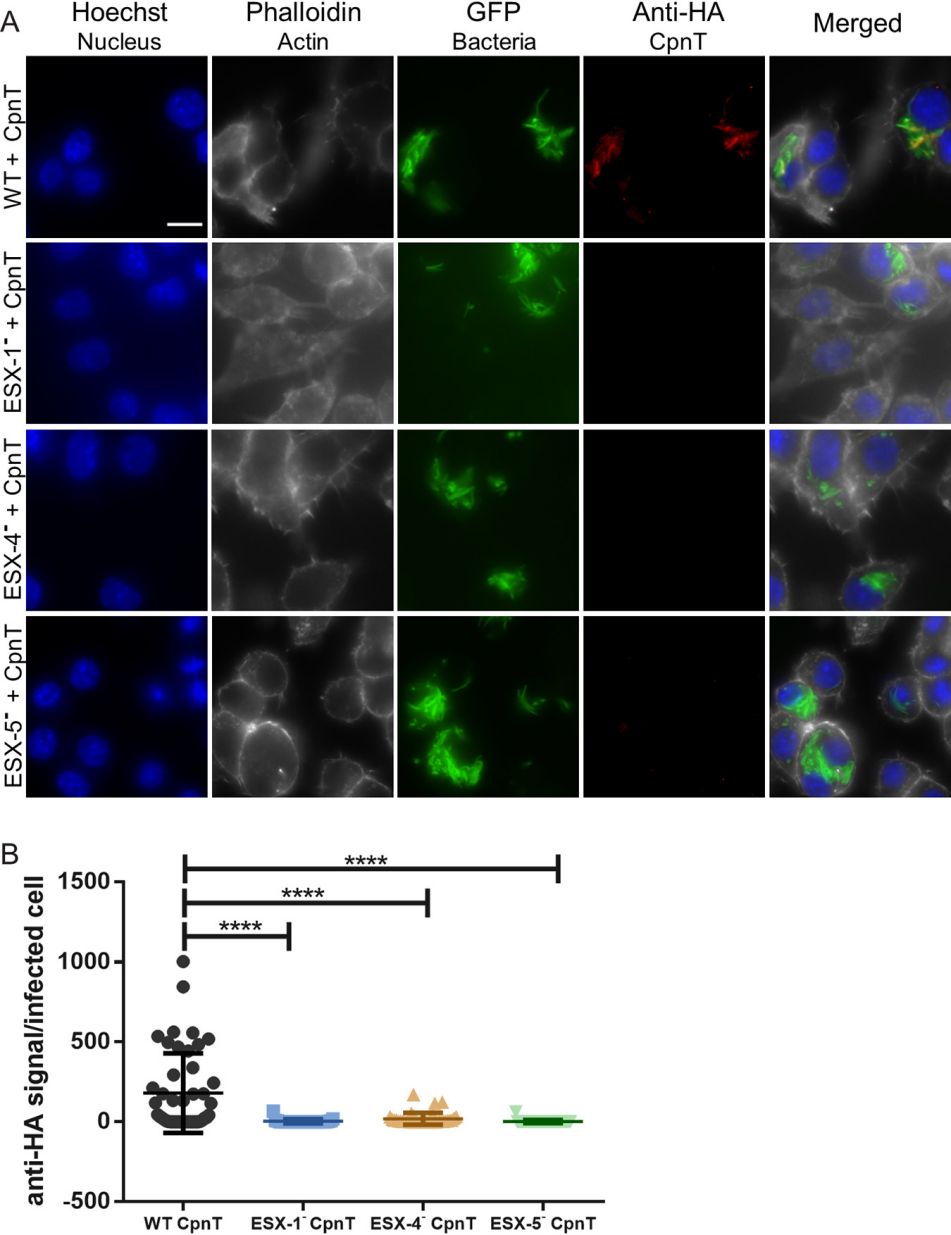
Secretion of CpnT in macrophages requires functional ESX-1, ESX-4, and ESX-5 systems. (A) Fluorescence microscopy of infected RAW 264.7 macrophages with WT, ESX-1-deficient (ESX-1^–^), ESX-4-deficient (ESX-4^–^), and ESX-5-deficient (ESX-5^–^) strains expressing *cpnT*. All strains expressed *gfp* from the L5 bacteriophage integration site. Macrophages were infected at an MOI of 2 and fixed at 24 h postinfection. Cells were permeabilized with 0.2% Triton X-100 and immunolabelled with the anti-HA antibody (CpnT-HA). Additional dyes were used to label the nuclei (Hoechst) and the actin filaments (Phalloidin). The scale bar represents 10 μm. Images are a representation of five biological replicates. (B) The fluorescence quantification of the anti-HA signal per infected RAW macrophage, from microscopy pictures, was calculated using automated image analysis by the software CellProfiler. Analysis was performed on one representative experiment out of three independent experiments with 30 to 46 infected cells. One-way ANOVA and multiple comparisons using Dunnet’s statistical test were performed for statistical significance. ****, *P* < 0.0001.

In order to investigate whether CpnT secretion was also dependent on ESX-5 during infection, we infected RAW macrophages with WT and the deficient strains of ESX-1, ESX-4, and ESX-5, each containing CpnT, and detected CpnT by immune labeling (CpnT-HA, anti-HA antibody) (Fig. 4A). The fluorescent signal originating from CpnT was quantified (Fig. 4B). Similar to our secretion analysis using axenic cultures, CpnT secretion was dependent on a functional ESX-5 secretion system during macrophage infections, as no CpnT signal could be detected in the ESX-5- strain (Fig. 4A and B). Surprisingly, in these infection experiments, CpnT could also not be detected in macrophages infected with the ESX-1-deficient strain (Fig. 4A and B). A functional ESX-1 secretion system is crucial for translocation of the mycobacteria from the phagosome to the cytosol in both M. marinum and M. tuberculosis (4, 44); thus, we hypothesized that a cytosolic localization is a requirement for CpnT secretion.

Even more surprising was that CpnT could also not be detected in the macrophages infected with the ESX-4-deficient strain (Fig. 4A and B). Fluorescent CpnT signal of the infected macrophages with ESX-4^–^ + CpnT was similar to the ESX-5^–^ + CpnT and ESX-1^–^ + CpnT strains.

### The ESX-4 secretion system is not involved in phagosomal escape.

Recently, ESX-4 was shown to play a prominent role in phagosomal membrane rupture in M. abscessus (27). However, the function of ESX-4 in M. marinum and M. tuberculosis within macrophage infection has not been elucidated yet. We wondered whether CpnT was not secreted during infection in the ESX-4-deficient strain because the bacteria remained in the phagosome, similar to the ESX-1^–^ strain.

In order to address this question, we compared the amount of WT mycobacteria that escaped the phagosome to the ESX-4-deficient strain. As a control we used the well-characterized ESX-1^–^ strain M^VU^. Host ubiquitinating enzymes can recognize surface proteins of bacteria that have escaped the phagolysosome and coat them with ubiquitin (Ub) in order to continue with autophagy processes. Previously, we used this approach to identify phagosomal escape of M. marinum using antibody FK2, which binds mono- and poly-ubiquitinated proteins (45). Macrophages were infected with *gfp*- and *cpnT*-expressing strains of WT, ESX-1, and ESX-4-deficient strains.

**FIG 5:**
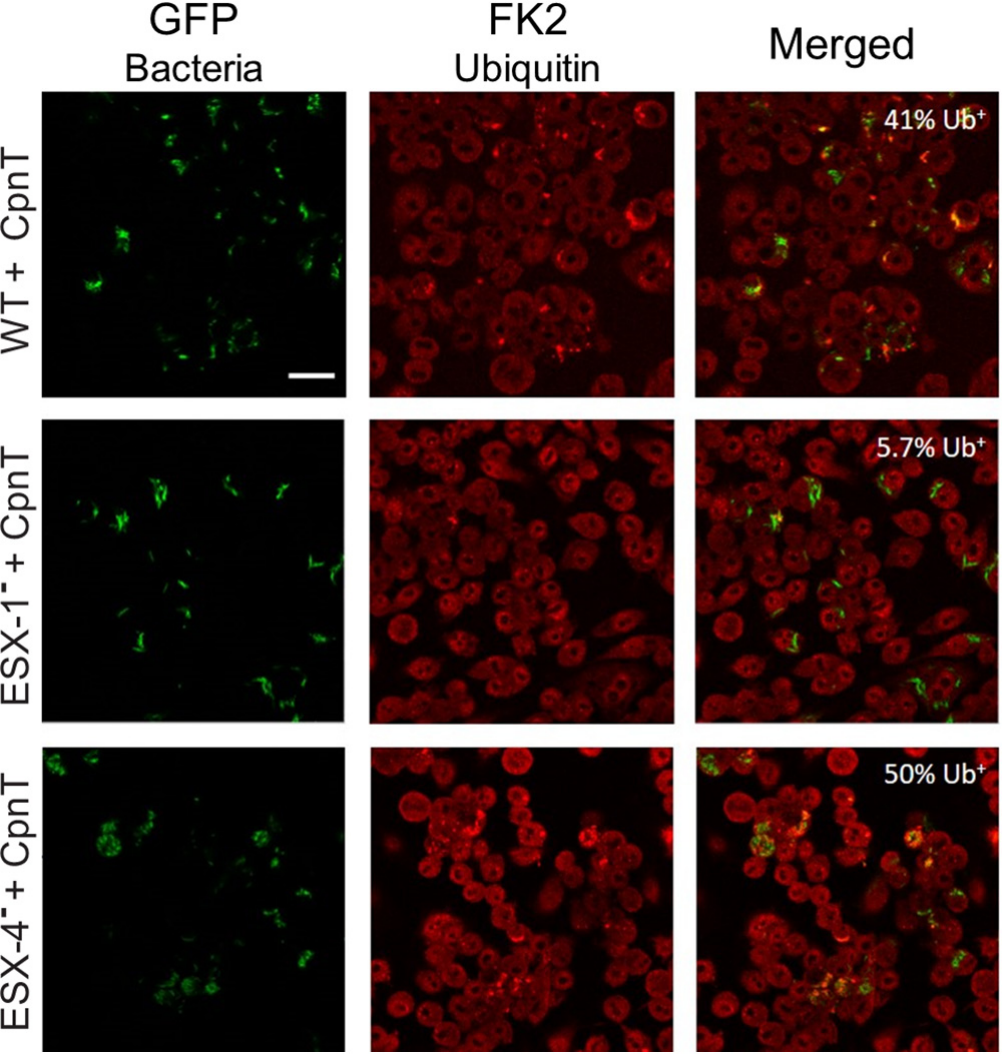
The ESX-4 system is not required for phagosomal escape. Detection of cytosolic ubiquinated mycobacteria (Ub^+^). Macrophages were infected with WT, ESX-1-deficient (ESX-1^–^), and ESX-4-deficient (ESX-4^–^) bacterial strains expressing *cpnT*. All strains expressed *gfp* from the L5 bacteriophage integration site. The infection conditions are described in Fig. 4. Cells were permeabilized and immunolabelled with anti-ubiquitin (FK2) antibody; images were obtained by confocal microscopy. WT+ CpnT *n* = 119, ESX-1^–^+ CpnT *n* = 70, ESX-4^–^+ CpnT *n* = 152 (*n* = number of bacteria). Colocalized signal of GFP and FK2 represent Ub^+^ bacteria (cytosolic bacteria). The proportion of Ub^+^ bacteria is displayed on the upper-right corner of each picture. The scale bar represents 20 μm.

Twenty-four hours postinfection, the number of cytosolic bacteria was determined by immune staining for ubiquitinated-coated bacteria (Fig. 5). As expected, macrophages infected with the ESX-1-deficient strain showed almost no bacteria that were labeled with the FK2 antibody (5.7% of bacteria were Ub^+^; Fig. 5), while in the case of the WT infection, almost half of the bacteria were positive (41%; Fig. 5). Infection with the ESX-4-deficient strain showed similar results as WT infection (50% of Ub^+^ bacteria; Fig. 5); thus, we could conclude that ESX-4 did not play a significant role in phagosome membrane rupture or phagosomal escape in M. marinum. This result also means that phagosomal escape is therefore not the reason for the observed lack of CpnT secretion by the ESX-4-deficient strain.

### Coinfection with WT bacteria restores CpnT secretion in ESX-1^−^ and ESX-4^−^ deficient strains.

**FIG 6:**
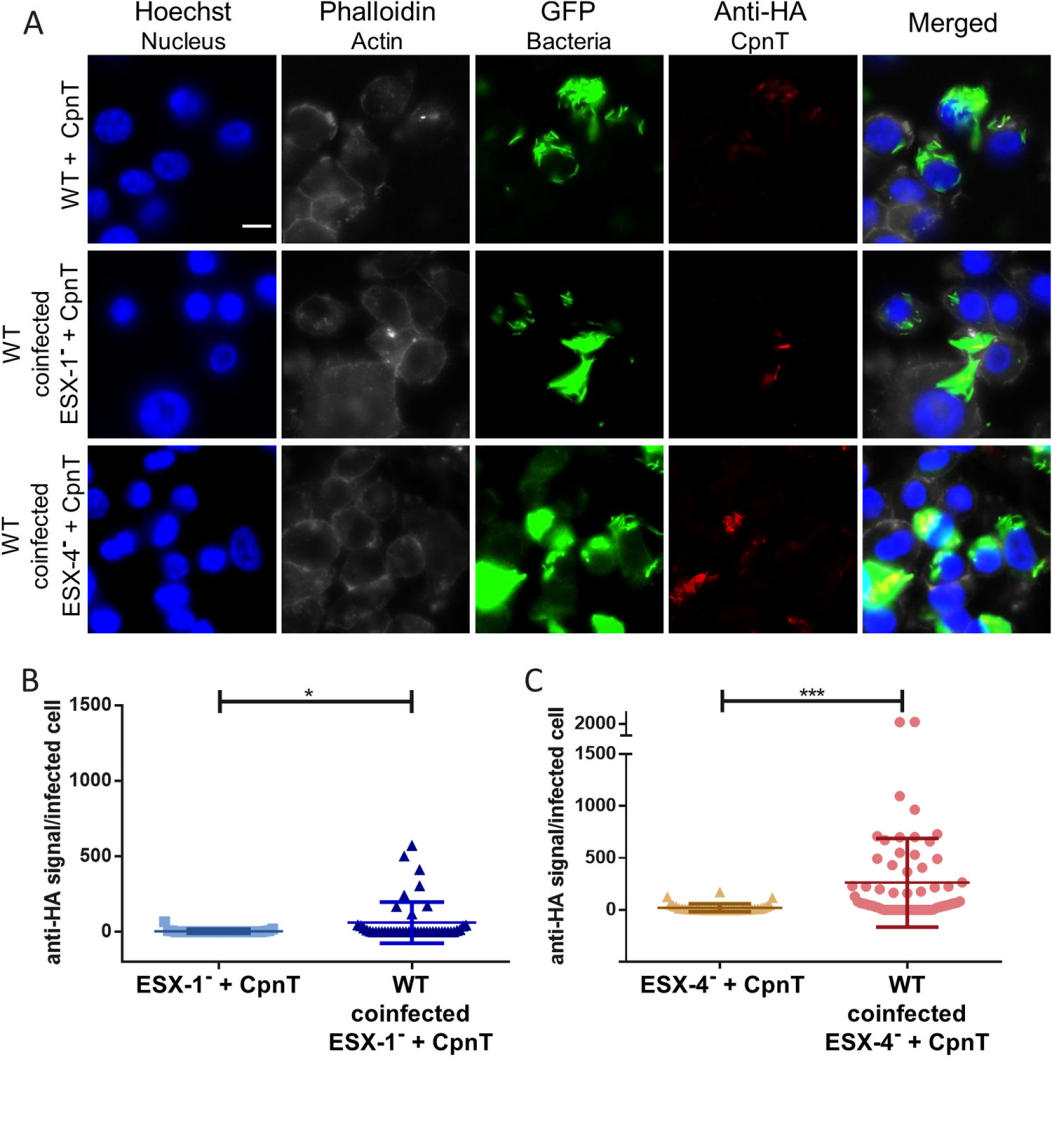
Coinfection with WT strain rescues CpnT secretion in ESX-1- and ESX-4-deficient strains within macrophages. (A) Fluorescence microscopy of RAW 264.7 cells coinfected with WT and either ESX-1^–^ or ESX-4^–^ strains expressing *cpnT*. WT bacteria expressing *cpnT* served as a positive control. All strains expressed *gfp*. Prior to the day of infection, WT cultures were mixed with the respective ESX mutant strain to ensure a heterogeneous culture. Macrophages were infected at an MOI of 5 and fixed at 24 h postinfection. Cells were permeabilized with 0.2% Triton X-100 and immunolabelled with the anti-HA antibody (CpnT-HA). Additional dyes were used to label the nuclei (Hoechst) and the actin filaments (Phalloidin). The scale bar represents 10 μm. (B and C) The fluorescence quantification of the anti-HA signal per infected RAW 264.7 macrophage, from microscopy pictures, was calculated using automated image analysis by the software CellProfiler. Analysis was performed on one representative experiment out of three independent experiments with a total number of infected macrophages between 30 and 60. A paired *t* test was performed for statistical significance. ***, *P* < 0.001; *, *P* < 0.05.

Fnally, we wanted to investigate whether the impaired secretion of CpnT in macrophages could be rescued by the presence of the WT strain. Cross-complementation would indicate that a secreted factor is required for this process; alternatively, we wanted to investigate whether ESX-1 and ESX-4 had a direct role in CpnT secretion inside macrophages. The ESX-1^–^ strain (ESX-1 deficient) expressing *cpnT* was cultured together with the WT prior to the day of infection to ensure that the bacteria would form heterogeneous microcolonies. We reasoned that if a WT bacterium, containing a functional ESX-1 system, was trapped in the same phagosome with a bacterium lacking this functional system, the WT bacterium would enable the mutant strain to translocate to the host cell cytosol and trigger CpnT secretion during infection. The macrophages were coinfected and immunolabelled in order to detect CpnT secretion (Fig. 6A). Microscopy revealed that, under these coinfection conditions increased amounts of CpnT could, indeed, be observed. Quantification of the CpnT signal (anti-HA) showed that the WT could significantly rescue the CpnT secretion (Fig. 6B), although again, strong heterogeneity was observed, similar to that shown previously for WT bacteria secreting CpnT. Even though cross-complementation was observed, the number of positive cells was lower than infected cells with WT+ CpnT alone (Fig. 4). Probably not all ESX-1^−^ bacteria were trapped together with WT bacteria and successfully escaped the phagosome together.

We performed the same coinfection studies in macrophages with WT and ESX-4^−^ (ESX-4 deficient) expressing *cpnT* (Fig. 6A). To our surprise, this mutant could also be rescued for CpnT secretion by the presence of WT bacteria, while the ESX-5^–^ (ESX-5 deficient) strain could not be rescued (Fig. S3) The fluorescence signal (anti-HA) of infected cells was quantified, and we observed again that with the WT strain, the mutant strain could secrete CpnT (Fig. 6C). Since the ESX-4-deficient strain was able to escape the phagosome by itself (Fig. 5), the mechanism by which WT bacteria rescue CpnT secretion remains obscure, but our results indicate that an ESX-4 secreted factor must play a role in this process.

10.1128/mBio.02983-20.3 FIG S3 Coinfection with the WT strain rescues CpnT secretion in ESX-1-, ESX-4-, and ESX-5-deficient strains within macrophages. (A) Fluorescence microscopy of RAW cells coinfected with WT and either ESX-1^–^, ESX-4^–^, or ESX-5^–^ strains expressing *cpnT*. WT bacteria expressing *cpnT* served as a positive control. All strains expressed *gfp*. Prior to the day of infection, WT cultures were mixed with the respective ESX mutant strain to ensure a heterogeneous culture. Macrophages were infected at an MOI of 5 and fixed at 24 h postinfection. Cells were permeabilized with 0.2% Triton X-100 and immunolabelled with the anti-HA antibody (CpnT-HA). The scale bar represents 10 μm. (B) The fluorescence quantification of the anti-HA signal per infected RAW macrophage, from microscopy pictures, was calculated using automated image analysis (CellProfiler). Analysis was performed on one representative experiment out of three independent experiments with a total number of infected macrophages between 20 and 30 cells. A paired *t* test was performed for statistical significance, ****, *P* < 0.0001; **, *P* < 0.01. Download FIG S3, JPG file, 0.9 MB. Copyright © 2021 Izquierdo Lafuente et al. 2021 Izquierdo Lafuente et al. https://creativecommons.org/licenses/by/4.0/ This content is distributed under the terms of the Creative Commons Attribution 4.0 International license.

## DISCUSSION

Previous studies have described CpnT (Rv3903c) as the first secreted toxin produced by M. tuberculosis (31–33). However, in these studies, the secretion pathway was not resolved. In mycobacteria, transport of virulence factors and specialized substrates is often mediated by one of the T7S systems (8). In this work, we were able to demonstrate that CpnT toxin of M. tuberculosis is an ESX substrate and specifically secreted through ESX-5.

CpnT shared some general characteristics with other ESX substrates, such as the secretion signal motif, YxxxD/E, directly following a helix-turn-helix domain (15). When this motif was mutated (Y88AxxxE, _AxxxE_CpnT), CpnT secretion was abolished in culture (Fig. 1C). This means that the secretion motif is required for efficient secretion, supporting the hypothesis of CpnT being an ESX substrate. The homology with the T7S substrate EspB extended beyond the first 100 amino acids (Fig. 1B). EspB is the only mycobacterial T7S substrate that forms a four-helix bundle instead of a helix-turn-helix and is therefore probably secreted as a monomer. Because we observed this extended homology, possibly also CpnT is secreted as a monomer.

A study from Sun et al. (33) showed that inside the M. tuberculosis-infected macrophages, the C-terminal domain of CpnT, the toxin, was found on the bacterial surface but also in the cytosol in specific spots, probably attached to vesicles. In our infection experiments, CpnT was predominantly located on the bacterial surface of M. marinum, and no punctuated staining was observed (Fig. 2). During secretion analysis in bacterial cultures, we also did not observe the processed band of the corresponding cleaved toxin, suggesting that the processing of this toxin was specific for M. tuberculosis (31) (Fig. 1C). If we combine these two observations, we hypothesize that within infected macrophages, the noncleaved form of CpnT stays attached to the bacterial surface, whereas the cleaved form in M. tuberculosis can associate with intracellular structures. Mutations in the secretion signal (_AxxxE_CpnT) resulted in an abrogated Cpnt secretion; however, infection experiments showed low CpnT signal when infecting with WT+ _AxxxE_CpnT. This was probably due to protein leakage, as _AxxxE_CpnT fluorescent signal quantification was minimal and not significantly different compared to infection with WT bacteria not expressing *cpnT* (Fig. 2A and B). Nevertheless, the AxxxE mutation did not affect the surface localization of CpnT, and therefore this ESX-5 substrate behaves differently than LipY and PE25 (15).

Next, we identified which ESX system is responsible for the secretion of CpnT. Notably, CpnT was not present in the culture supernatant of the ESX-5-deficient strain, and its secretion was restored by complementation (Fig. 1C). Moreover, looking at infected macrophages, secretion of CpnT was not detected (Fig. 4). With all these findings, we could conclude that CpnT is an ESX-5 substrate. Identifying CpnT, a recently discovered toxin, as an ESX-5 substrate supports our previous finding that ESX-5 mutants of both M. marinum and M. tuberculosis showed significantly reduced macrophage cell death (21). In the same study, we showed that this effect of ESX-5 activity on macrophage cell death was not caused by an inability to escape the phagosome.

Interestingly, the secretion characteristics of CpnT showed major differences during infection. Whereas in culture we only observed abolished CpnT secretion in an ESX-5-deficient strain, during infection experiments, the ESX-1- and ESX-4-deficient mutants also showed no secretion of CpnT (Fig. 1C and 4). To make sure we could identify all intracellular and intraorganelle secreted CpnT, we used Triton X-100 during staining procedures, as it permeabilized all lipid bilayers of the host cell (46). Therefore, our data indicate that in M. marinum, CpnT is either degraded in the phagosome by host enzymes or is not exported to the cell surface in the ESX-1-deficient strain. Inside the macrophage, the ESX-1 system is necessary for mycobacteria to rupture the phagosome (4, 44). Possibly, due to the high toxicity of CpnT, its expression and secretion need to be tightly regulated during infection. Hence, we considered that CpnT can only be released when bacteria have successfully escaped the phagosome. This is in line with the previous observation that CpnT-producing M. tuberculosis has to rupture or escape the phagosome before it can kill the macrophage by depleting NAD^+^ pools (33). Cross-complementation of an ESX-1-deficient strain expressing *cpnT* with a wild-type strain lacking *cpnT* confirmed this hypothesis (Fig. 6). However, we cannot exclude that the influence of ESX-1 on CpnT secretion could be due to another ESX-1 substrate that is not required for phagosomal rupture.

**FIG 7:**
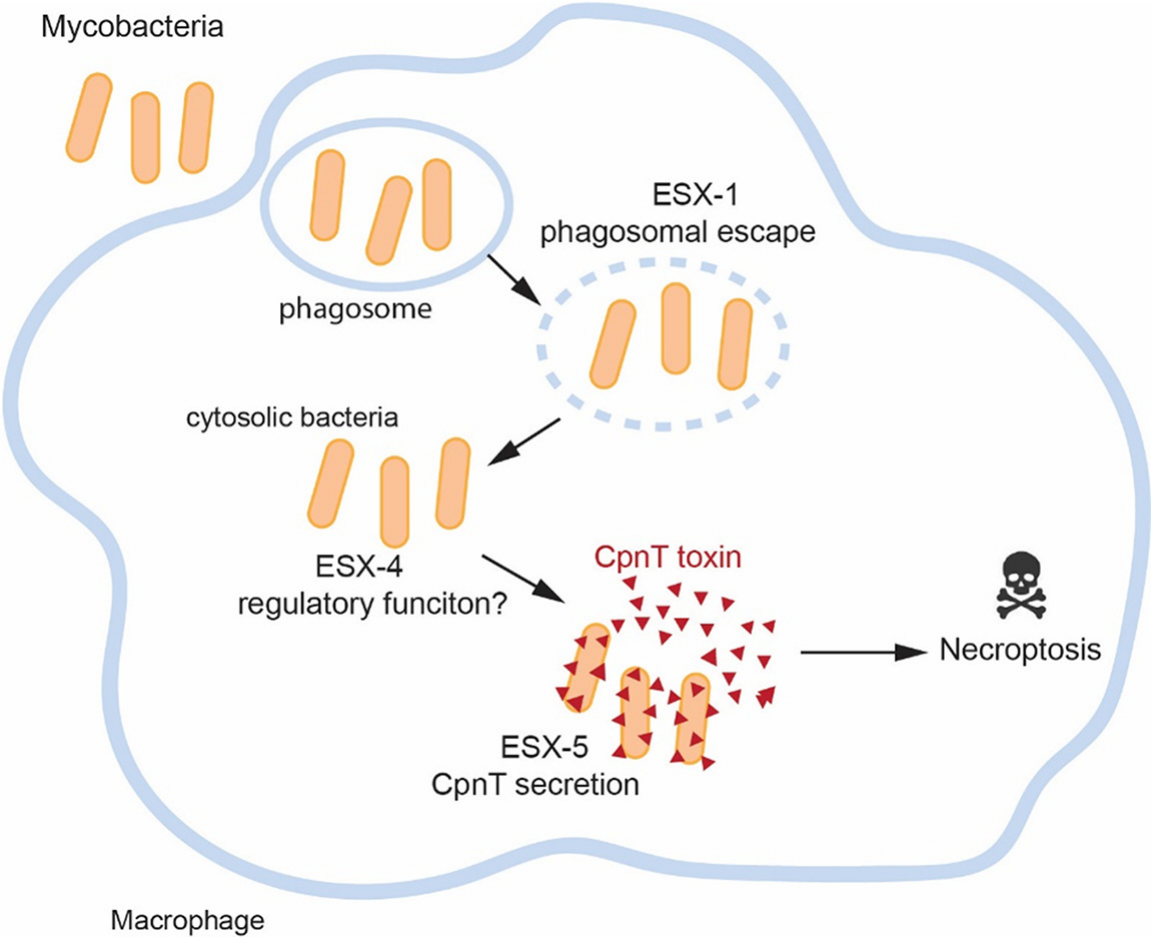
Model of CpnT secretion in mycobacterium-infected macrophages. Bacteria are phagocytosed by the macrophage and trapped in a phagosome to be eventually neutralized. Mycobacteria utilize the ESX-1 secretion system to escape from this phagosome to the cytosol. Once bacteria reach the cytosol, the ESX-4 system could sense the cytosolic environment and then activate the production of virulent factors. CpnT is then secreted by the ESX-5 secretion system to kill the macrophage.

The most striking result was that macrophages infected with an ESX-4-deficient strain did not display any CpnT-HA secretion (Fig. 4). The role of ESX-4, the most ancient ESX system, is still not very clear. In the fast-growing pathogen M. abscessus, ESX-4 was shown to be involved in phagosomal rupture (27). However, we demonstrated in this study that ESX-4 of slow-growing M. marinum is not required to rupture the phagosome (Fig. 5). We have shown that, just like ESX-1, ESX-4 is also indirectly linked to CpnT secretion, as we could cross-complement this secretion defect with WT bacteria. In contrast, it was not possible to complement the ESX-5-deficient mutant using coinfection experiments with WT bacteria (Fig. S3). Previous research has shown that most ESX-4 genes in M. tuberculosis are regulated by SigM, an alternative sigma factor (47). Interestingly, the SigM regulon also contains *esxE* and *esxF* genes (48), which not only are located in the same locus and possibly the same operon as *cpnT*, but we also demonstrated that the expression of *esxE* and *esxF* was necessary for CpnT secretion. The activation of the SigM regulon during infection and consequently the ESX-4 system could reveal an unexpected function of this system in virulence. It is not the first time that ESX-1 and ESX-4 systems have seemed to cooperate. M. smegmatis employs both ESX-1 and ESX-4 to promote a unique form of chromosomal DNA conjugation (26). We could also have a combined function of ESX-1 and ESX-4 in M. marinum, in which CpnT expression or secretion in macrophages only occurs when both ESX-1 and ESX-4 are present. Bacteria use virulence factors at different stages of the infection cycle. Sensing the environmental changes after translocation to the cytosol is critical for the regulation of many of these effector proteins (49). The ESX-4 system, possibly together with some ESX-1 substrates, could participate in the sensing of a certain intracellular signal or bacterial communication required for the regulation of CpnT expression and/or secretion. In our model (Fig. 7), we display our current hypothesis of how ESX-1 and ESX-4 are indirectly involved in intracellular CpnT secretion. Mycobacterial infection of host cells can trigger a cascade of molecular events that cause necroptosis and ultimately lead to lesion formation; individual ESX systems participate during this process to ensure full virulence. ESX-1 helps the bacteria to escape the phagosome, and we speculate that cytosolic bacteria could sense the new environment in the cytosol by ESX-4 or ESX-4 substrates. This system, possibly through the activation of SigM, would then lead to the secretion of CpnT by the ESX-5 system. Understanding the intracellular life cycle of M. tuberculosis will probably require a better-integrated understanding of multiple and perhaps even all ESX secretion systems.

## MATERIALS AND METHODS

### Bacterial strains and cell cultures.

Mycobacterium marinum M^USA^ was used as the parental WT strain, and its derivatives are listed in Table S1. All M. marinum strains were routinely grown at 30°C in liquid Middlebrock (Difco-BD Biosciences) 7H9 or solid Middlebrock 7H10 medium supplemented with 10% (vol/vol) ADS (5% [wt/vol] albumin, 2% [wt/vol] dextrose, and 0.16% [wt/vol] NaCl), 0.2% glycerol, and 0.01% (vol/vol) tyloxapol. Escherichia coli Dh5α, which was used for plasmid constructions, was grown in Luria-Bertani (LB) liquid and solid medium at 37°C. If necessary, antibiotics were added to the medium at the following concentrations: kanamycin (Kan, 25 μg ml^−1^; Sigma) and hygromycin (Hyg, 50 μg ml^−1^; Roche).

10.1128/mBio.02983-20.5 TABLE S1 Strains used in this study. Download Table S1, DOCX file, 0.01 MB. Copyright © 2021 Izquierdo Lafuente et al. 2021 Izquierdo Lafuente et al. https://creativecommons.org/licenses/by/4.0/ This content is distributed under the terms of the Creative Commons Attribution 4.0 International license.

### Molecular cloning.

All primers and plasmids used in this study can be found in Table S2 and Table S3, respectively. The plasmids in this study were constructed using the standard molecular cloning techniques summarized in Fig. S4. The integrative plasmid pML1337 (Kan^r^) was used for generating green fluorescent protein (GFP)-fluorescent bacteria (50). The integrative plasmid pMV::*eccC_5_mmar* was used for complementing the ESX-5-deficient strain (20). The *eccC_4_* deletion strain was constructed in an M. marinum WT M^USA^ strain background by homologous recombination as previously described (51). The homologous regions upstream and downstream of *eccC4* (*mmar_1102*) were constructed using primer pairs R101/R102 and R103/R104, respectively. A double-crossover event was confirmed by PCR using primers R105-R108. The introduced hygromycin resistance cassette was removed by transient expression of the gene *tnpR* to yield the strain M^USA^ *eccC4::res*.

10.1128/mBio.02983-20.4 FIG S4 Cloning strategy for new plasmids used in this study. Constructed plasmids are displayed in grey boxes. The primer pairs used for PCR amplification and the corresponding restriction enzymes for subsequent cloning are listed in white boxes. The DNA template for the PCR reactions is listed above the primer pairs. If several primer pairs are listed, overlap PCR was used to fuse the PCR fragments. When a DNA fragment was obtained by digestion of a plasmid, the utilized restriction enzymes and the length of the obtained fragments are indicated. Constructed plasmids with their features and primers with their sequences are listed in Tables S2 and S3. Download FIG S4, JPG file, 0.1 MB. Copyright © 2021 Izquierdo Lafuente et al. 2021 Izquierdo Lafuente et al. https://creativecommons.org/licenses/by/4.0/ This content is distributed under the terms of the Creative Commons Attribution 4.0 International license.

10.1128/mBio.02983-20.6 TABLE S2 Primers used in this study. Download Table S2, DOCX file, 0.01 MB. Copyright © 2021 Izquierdo Lafuente et al. 2021 Izquierdo Lafuente et al. https://creativecommons.org/licenses/by/4.0/ This content is distributed under the terms of the Creative Commons Attribution 4.0 International license.

10.1128/mBio.02983-20.7 TABLE S3 Plasmids used in this study. Download Table S3, DOCX file, 0.01 MB. Copyright © 2021 Izquierdo Lafuente et al. 2021 Izquierdo Lafuente et al. https://creativecommons.org/licenses/by/4.0/ This content is distributed under the terms of the Creative Commons Attribution 4.0 International license.

### Protein structure prediction.

The CpnT N-terminal structure was predicted based on homology modeling using Phyre2 (Protein Homology/AnalogY Recognition Engine; 35). The EspB protein from Mycobacterium smegmatis (Protein Data Bank accession number 4wj2) was used as a template. The three-dimensional (3D) structure was visualized and alignment of the N terminus of CpnT with EspB was carried out by structural superposition with the software PyMOL2. Root-mean-square deviation (RMSD) was used as a quantitative tool for measuring the distance between the atoms of the superposed structures.

### Protein secretion analysis and Genapol extraction.

Proteins from the supernatant were precipitated as described before (15). M. marinum strains were grown until the mid-logarithmic phase (optical density at 600 nm [OD_600_], 0.8 to 1.2) in 7H9-ADS-glycerol-tyloxapol liquid medium supplemented with appropriate antibiotics. Bacteria were washed and diluted to an OD_600_ of 0.35 to 0.4 in medium without ADS. After 16 h of growth, cells were harvested (3,000 × *g*, 5 min) and pellet and supernatant fractions were collected. Supernatants were precipitated with 100% trichloroacetic acid (TCA). Intact bacteria of the pellet fraction were incubated with 0.5% Genapol X-080 in phosphate saline buffer (PBS) for 30 min with head-over-head rotation at room temperature. The bacterial pellet and the Genapol-extracted bacteria were lysed by bead beating (100-μm silica beads; Biospec) to obtain the whole-cell lysate and the Genapol pellet fraction, respectively. Proteins were denatured with SDS, separated by SDS-PAGE, and detected by Western blotting.

### Bacteria subcellular fractionation.

Bacteria were grown until the mid-logarithmic phase (OD_600_, 0.8 to 1.2) in 7H9-ADS-glycerol-tyloxapol liquid medium supplemented with appropriate antibiotics. Cells were washed and diluted to an OD_600_ of 0.35 to 0.4. After 16 h of growth, cells were harvested (3,000 × *g*, 5 min), and pellets were resuspended in PBS. Bacteria were lysed by sonication on ice for 2.5 min, followed by incubation with lysozyme (10 mg ml^−1^), DNase (1 U), and phenylmethylsulfonyl fluoride (PMSF, 200 mM) for 30 min at 37°C. Sonication was repeated to ensure total lysis. Aliquots were taken for the whole-cell lysate fraction. Samples were next centrifuged at 3,000 × *g* for 5 min at 4°C. Supernatants were collected and transferred to special tubes for ultracentrifugation. Tubes were centrifuged at 120,000 × *g* for 30 min at 4°C. Supernatants were again collected and transferred to new tubes for ultracentrifugation. Pellets were resuspended in PBS. The ultracentrifugation step was repeated for pellet and supernatant fractions. The final samples were subjected to Western blot analysis.

### RAW 264.7 macrophage infection.

RAW 264.7 (ATCC TIB-71) macrophages were grown in Dulbecco’s modified Eagle medium (DMEM; Thermo Fisher) supplemented with 10% fetal bovine serum (FBS) at 37°C and 5% CO_2_. Cells were seeded (0.25 million cells ml^−1^) in a 12-well plate with glass coverslips and incubated overnight. For infection, bacteria in the mid-logarithmic phase, were diluted to an OD of 0.35 to 0.4 prior to the day of infection and grown overnight at 30°C. Bacteria were washed with PBS, and macrophages were infected at multiplicity of infection (MOI) of 2 and incubated at 33°C and 5% CO_2_. At 3 h postinfection (p.i.), cells were incubated with DMEM containing gentamycin (50 μg ml^−1^) for 1 h. Cells were then washed, and fresh medium was added. At 24 h p.i., the infected macrophages were fixed with 4% (vol/vol) paraformaldehyde (PFA) for 20 min at room temperature (RT) and subsequently washed three times and left at 4°C for immune labeling.

### Coinfection in RAW 264.7 macrophages.

Bacteria were cultured for 3 to 4 days until the mid-logarithmic phase as described before. Prior to the day of infection, bacteria were washed one time with PBS, and then the WT strain was mixed with the corresponding ESX mutant strain to a final total OD of 0.4 (OD = 0.2 of each strain) in the same culture. Infection was carried out as described in the previous methods. Macrophages were infected at an MOI of 5.

### Inmuno-labeling.

Fixed macrophages were incubated with 0.1 M glycine for 10 min and washed two times with PBS. Cells were permeabilized with 0.2% (vol/vol) Triton x-100 for 10 min and later washed two more times with PBS. For blocking, cells were incubated for 1 h at RT with 1% (wt/vol) bovine serum albumin (BSA; Sigma) in PBS. The first antibody, anti-HA.11 (1:500; Covance), was diluted in blocking buffer (0.1% BSA in PBS), and cells were incubated for 2 h at RT. After three washes with PBS, secondary anti-mouse Alexa Fluor 568 (1:200; Invitrogen), Hoechst 33342 dye (1:1,000; Invitrogen), and phalloidin (1:400; Invitrogen) were incubated with cells for 30 min in darkness. Cells were then washed three times with PBS, and coverslips were mounted (mounting medium, Vector Laboratories) and imaged on an Olympus IX83 microscope or Nikon confocal microscope.

### Western blot analysis.

Proteins were separated by SDS-PAGE (12.5% polyacrylamide) and transferred to a nitrocellulose membrane (GE Healthcare Life Sciences). Membranes were blocked for 1 h at RT. Proteins were labeled with primary mouse monoclonal antibodies anti-HA.11 (1:5,000), anti-EsxA (1:500, Hyb 76-8; Statens Serum Institut, Copenhagen, Denmark), anti-PE_PGRS (1:10,000,7C4.1F7) (5), anti-EccB_5_ (1:5,000) (4), and anti-GroEL (1:10,000, CS44; John Belisle, NIH, Bethesda, MD, USA) for 1 h at RT. Secondary antibody goat anti-mouse IgG (1:2,500; Rockland) conjugated with horseradish peroxidase (HRP) was incubated for 1 h at RT, and proteins were visualized with ECL substrate (GE Healthcare Life Sciences). Protein concentrations were determined using the bicinchoninic acid assay (BCA assay; Thermo Scientific) for a proper normalization during loading.

### Fluorescence quantification and statistical analysis.

Fluorescent images were taken of infected RAW 264.7 macrophages with mycobacteria. The fluorescence intensity was calculated by using CellProfiler 3.1.9. Scatterplots were made using Graph Pad Prism 6.01. Error bars represent the standard error of the mean. A one-way analysis of variance (ANOVA) was performed, followed by Dunnett’s multiple-comparison test to analyze statistical significance compared to the control sample, WT+ CpnT. A *t* test was also performed when comparing the mean of only two samples to analyze statistical significance. Statistical significance is shown as ns, *P* > 0.05; *, *P* ≤ 0.05; **, *P* ≤ 0.01; ***, *P* ≤ 0.001; ****, *P* ≤ 0.0001.
