# Supplementary figures and images for: Mycobacterium tuberculosis Toxin CpnT Is an ESX-5 Substrate and Requires Three Type VII Secretion Systems for Intracellular Secretion
Source: mBio. 2021 Mar 2;12(2):e02983-20. doi: 10.1128/mBio.02983-20 (PMC8092274; doi:10.1128/mBio.02983-20)

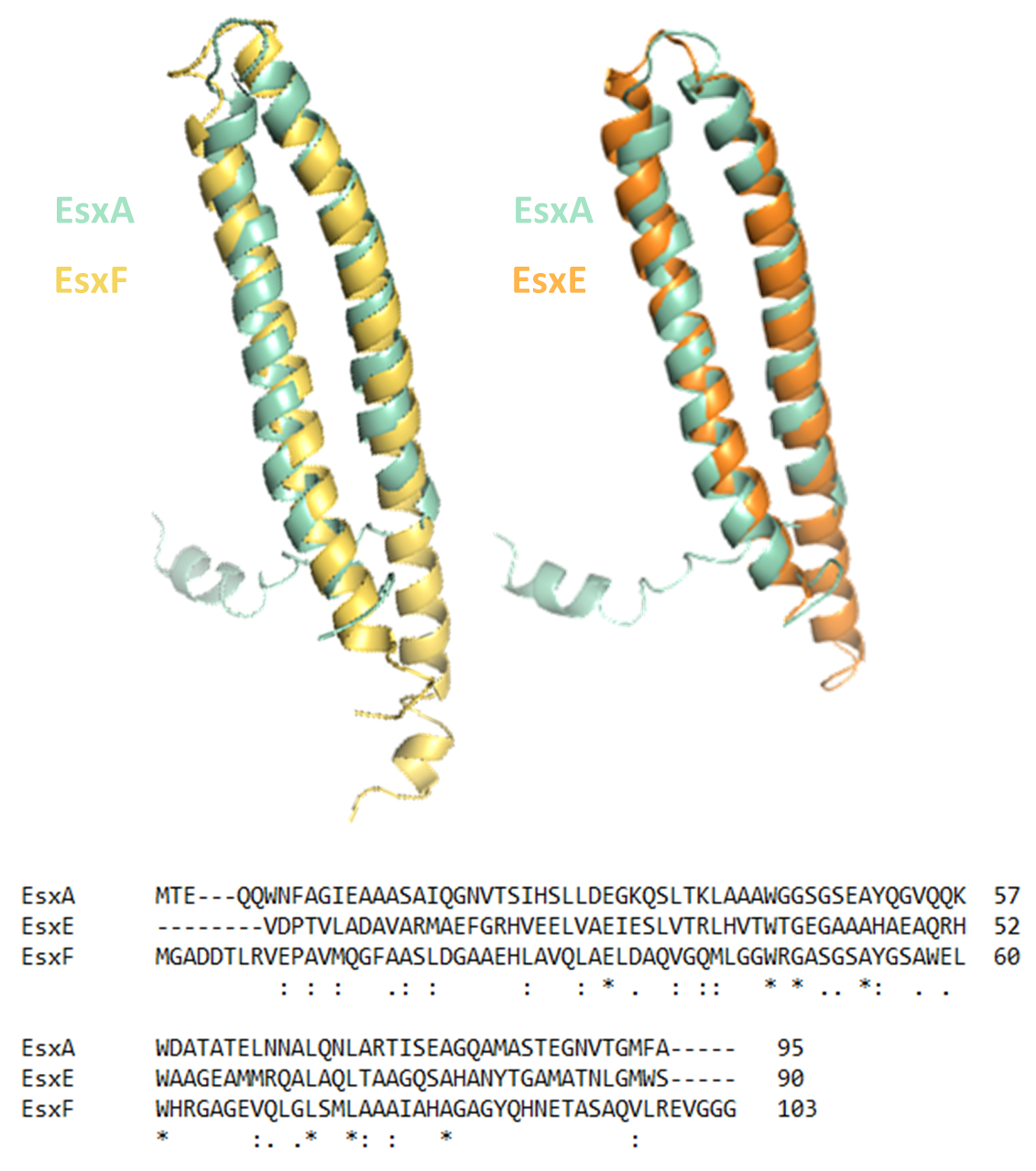

Supplement: FIG S1 [file mBio.02983-20-sf001.jpg]

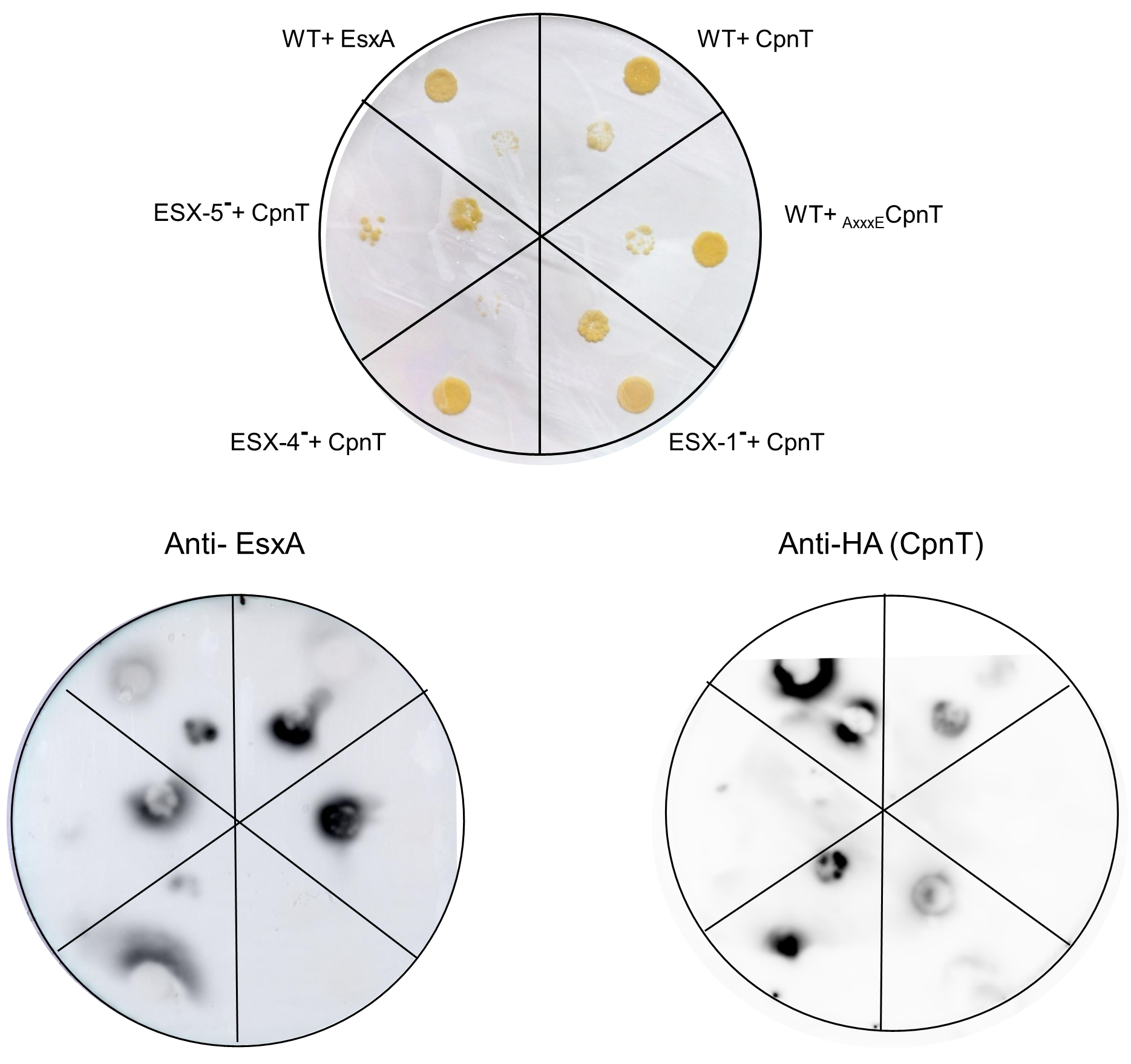

Supplement: FIG S2 [file mBio.02983-20-sf002.jpg]

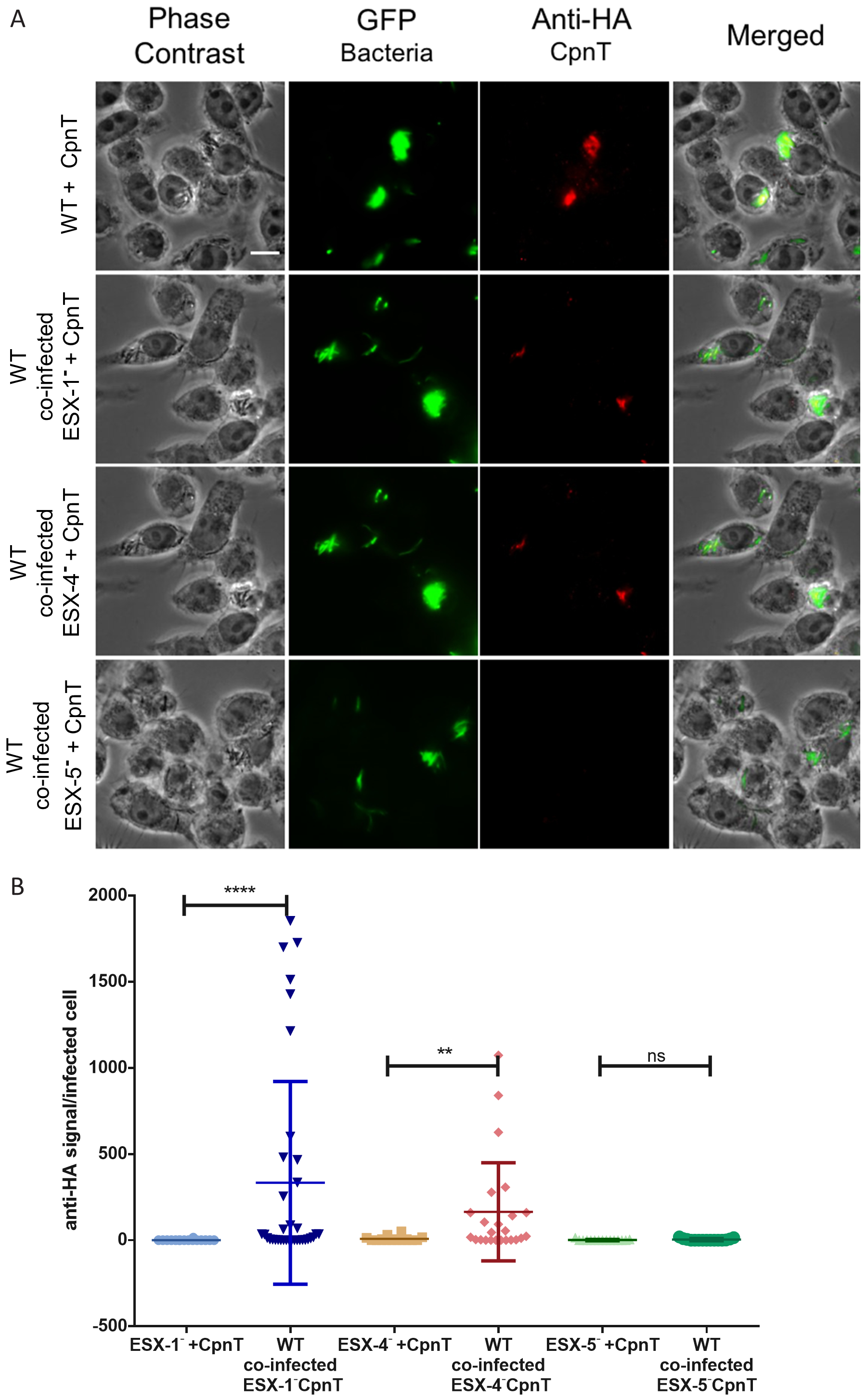

Supplement: FIG S3 [file mBio.02983-20-sf003.jpg]

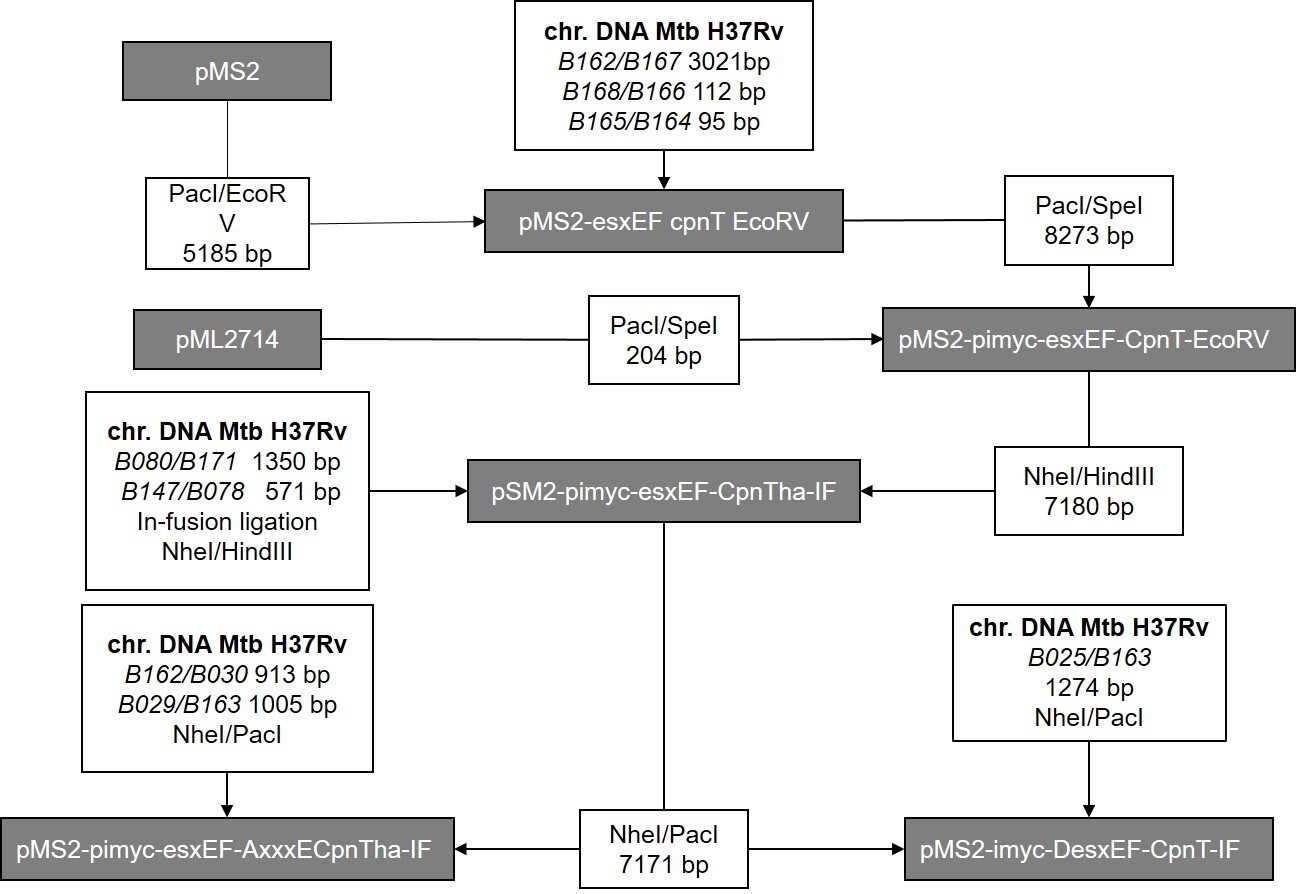

Supplement: FIG S4 [file mBio.02983-20-sf004.jpg]
