# Supplementary material for: Mycobacterium tuberculosis Toxin CpnT Is an ESX-5 Substrate and Requires Three Type VII Secretion Systems for Intracellular Secretion
Source: mBio. 2021 Mar 2;12(2):e02983-20. doi: 10.1128/mBio.02983-20 (PMC8092274; doi:10.1128/mBio.02983-20)
Supplement: TABLE S1 [file mBio.02983-20-st001.docx]

| **Strains** | **Characteristics** | **Referred to as** | **References** |
| --- | --- | --- | --- |
| M^USA^ | *M. marinum* WT strain | WT | (8) |
| M^VU^ | *M. marinum* M^USA^ *eccCb1* frame shift mutation | ESX-1^-^ | (5) |
| M^USA^ ∆*eccC4* | M^USA^ *eccC_4_::res* | ESX-4^-^ | This study |
| M^USA^ ∆*eccC5* | M^USA^ *eccC_5_::loxP, pSMT3::mspA strep^R^* | ESX-5^-^ | (37) |
