# Supplementary material for: Mycobacterium tuberculosis Toxin CpnT Is an ESX-5 Substrate and Requires Three Type VII Secretion Systems for Intracellular Secretion
Source: mBio. 2021 Mar 2;12(2):e02983-20. doi: 10.1128/mBio.02983-20 (PMC8092274; doi:10.1128/mBio.02983-20)
Supplement: TABLE S2 [file mBio.02983-20-st002.docx]

| **Oligonucleotide** | **Sequence 5’-3’** |
| --- | --- |
| B025 | CGCATGCTTAATTAAGGGAGAACATGGCGCCGTTGGCGGTCGA |
| B029 | ATGAGCGCGCACAACGCCTCGTTGGCCGAGGC |
| B030 | GCCTCGGCCAACGAGGCGTTGTGCGCGCTCAT |
| B078 | CAATTAATTAGCTAAAGCTTAACCCTTATAGTCCTTCCAAAGGG |
| B080 | GCTAGGCAAGACCGTATTCG |
| B147 | CGTACGACGTGCCGGACTACGCGTGACAATAGGTGTGGACCTGTC |
| B162 | ATGCTTAATTAACAGAAAGGAGGATTTCAACTATCATGGGTGCCGACGACACGCT |
| B163 | CAAAGCTAGCGTCGCGGCGT |
| B164 | TAGCGATATCCTGTCGCAACACCCCGCGC |
| B165 | CGGATCCATCAAAGTGCGGATA |
| B166 | TATCCGCACTTTGATGGATCCG |
| B167 | ATGGTATACGCGTTGTAGGGGTCG |
| B168 | CGACCCCTACAACGCGTATACCAT |
| B171 | GGCACGTCGTACGGGTAGATATCCTGTCGCAACACCCCGCGCC |
| R101 | TTTTTTTTGCATCTTTTGCGTCGATGACCACCGCTGTCC |
| R102 | TTTTTTTTGCATAAATTGCATCGCGGCGAGCATCAGATT |
| R103 | TTTTTTTTGCATTTCTTGCGACCGTGCAACACAGCCATT |
| R104 | TTTTTTTTGCATAGATTGCTCTCAGCGAGTCCGAGTACC |
| R105 | GCACATGACGGCGGTGAAGG |
| R106 | AGGATCCAGGACCTGCCAAT |
| R107 | AGGGCGGCGCTACATTTCTC |
| R108 | GGTGTTGGGCAGCTCAAAGG |
