# Supplementary material for: Mycobacterium tuberculosis Toxin CpnT Is an ESX-5 Substrate and Requires Three Type VII Secretion Systems for Intracellular Secretion
Source: mBio. 2021 Mar 2;12(2):e02983-20. doi: 10.1128/mBio.02983-20 (PMC8092274; doi:10.1128/mBio.02983-20)
Supplement: TABLE S3 [file mBio.02983-20-st003.docx]

| **Plasmids** | **Characteristics** | **References** |
| --- | --- | --- |
| pMS2-esxEF-cpnT EcoRV | ColE1 origin; PAL5000 origin; *esxEF-*_H792N,Q822K_*cpnT*, *hyg;* 8351 bp | This study |
| pMS2-pimyc-esxEF-cpnT-EcoRV | ColE1 origin; PAL5000 origin; p_imyc_ *esxEF-* _H792N,Q822K_*cpnT*, *hyg;* 8483 bp | This study |
| pSM2-pimyc-esxEF-CpnT-HA-IF | ColE1 origin; PAL5000 origin; p_imyc_ *esxEF-* _H792N,Q822K_*cpnT-HA-rv3902c*, *hyg;* 9038 bp | This study |
| pMS2-pimyc-esxEF-AxxxEcpnTHA-IF | ColE1 origin; PAL5000 origin; p_imyc_ *esxEF-* _Y88A_,_H792N,Q822K_*cpnT*-HA*-rv3902c*, *hyg;* 9038 bp | This study |
| pMS2-imyc-CpnT-IF | ColE1 origin; PAL5000 origin; p_imyc_ _H792N,Q822K_*cpnT*-HA*-rv3902c*, *hyg;* 8424 bp | This study |
| pMV::*eccC5mmar* | p_hsp60_ *mmar2665* int, L5 *attP*; *aph*, oriE; 4167bp | (20) |
| pML1337 | p_smyc_ *mycgfp2+*, int, L5 *attP*; *aph*, oriE, 5101 bp | (50) |
| pMS2 | ColE1 origin; PAL5000 origin; *hyg;* 5229 bp | (52) |
| pML2714 | pUC origin; pAL5000ts; p_groEL_-*cre*; p_imc_-*pamcherry1m*; *aph*; 7613 bp | (53) |
| pSMT3::*esxB_1/esxA_1* WT.HA | p_hsp60_ *mmar0187 mmar0188-HA*, PAL5000, *hyg;* 568 bp | (37) |
